# Digital Health Evaluation of Canadians With Physical Disabilities Compared to Those Without Chronic Conditions

**DOI:** 10.7759/cureus.96324

**Published:** 2025-11-07

**Authors:** Vic DaSilva, Julien M Meyer, Pria Nippak, Housne Begum

**Affiliations:** 1 Health Services Management, Ted Rogers School of Management, Toronto Metropolitan University, Toronto, CAN

**Keywords:** canada, digital health, interoperability, physical disability, virtual care

## Abstract

Background: Nearly 8.0 million Canadians have one or more disabilities, and this population uses a disproportionate portion of healthcare resources. Digital health, including virtual care and health information interoperability, offers specific benefits but also specific challenges to patients with physical disabilities (PwPD).

Objective: This study examined the utilization of virtual care and digital health access among PwPD compared to those without chronic conditions (NCC) by examining the usage rates, preferred modalities, the impact of interoperability, and the role of access to personal digital health information.

Methods: A cross-sectional survey, termed the 2022 Canadian Digital Health Survey (CDHS), was conducted to target any Canadians over 16 who have used healthcare in Canada over the last 12 months.

Results: This study compared the PwPD group (n=674) with the NCC group (n=5273). PwPD were significantly older and had higher digital health service utilization among PwPD compared to NCC individuals. They reported higher engagement in digital health services like prescription management, telephone consultations, and remote patient monitoring. Healthcare system coordination challenges were more prevalent with 24.4% experiencing care delays and 31.8% reporting frustration. PwPD reported positive outcomes in accessing personal health information (PHI), setting health goals, avoiding doctor visits, and preventing ER visits.

Conclusion: This study highlights the link between digital health quality and care outcomes in Canadian healthcare. It reveals a preference for telephone consultations in virtual care, emphasizing the importance of accessible technology. Future studies need to be completed to better understand people with PwPD and the larger group of patients with chronic conditions.

## Introduction

In Canada, individuals with disabilities account for 27% of the population, representing nearly 8.0 million people as of 2022, a 5% increase since 2017 [1]. This demographic disproportionately utilizes healthcare resources, with Canadians with disabilities relying on healthcare services at a higher rate than the general population [2]. Despite their reliance on the healthcare system, individuals with disabilities remain underrepresented in research, limiting insights into their healthcare experiences and outcomes [3].

Digital health, defined as the integration of technology into healthcare systems to enhance the delivery, accessibility, and management of health services, has become increasingly vital in addressing modern healthcare challenges [4]. It encompasses a wide range of technological tools, including electronic health records (EHRs), telemedicine platforms, wearable health monitors, and mobile applications. These innovations hold the potential to improve healthcare delivery by enabling remote monitoring, reducing administrative burdens, and fostering personalized care [5]. By empowering patients to take an active role in their health management, digital health has proven to be a transformative force, particularly for individuals with disabilities.

Virtual care, a subset of digital health, has demonstrated effectiveness and high patient satisfaction, particularly for patients with disabilities (PwD) [5,6]. Reduced travel and wait times are especially advantageous for patients with physical disabilities (PwPD), whose mobility challenges can impede access to traditional in-person care. The COVID-19 pandemic accelerated the adoption of virtual care, with studies showing that it is comparable in effectiveness to in-person visits and can reduce emergency room follow-ups [7,8]. This shift underscored the potential of virtual care to address long-standing access barriers while meeting the urgent needs of patients during a global crisis [9].

Despite its benefits, virtual care poses unique challenges for PwPD. Many individuals face technological barriers, including difficulties navigating digital platforms, low digital literacy stemming from socioeconomic disparities, and privacy concerns [10,11]. Age is another factor influencing adoption, with younger individuals more likely to engage with virtual care services compared to older adults [12]. These challenges contribute to disparities in virtual care utilization and outcomes, emphasizing the need for tailored solutions to ensure equitable access [13]. Additionally, the pandemic highlighted these disparities, as some PwD experienced greater difficulty accessing necessary healthcare services despite the broader availability of virtual care [9,14].

Digital health interoperability, defined as the seamless integration of health information across various platforms and providers, has emerged as a pivotal element in enhancing the quality and efficiency of healthcare delivery. However, significant gaps remain, including limited clinician willingness to adopt digital health solutions and fragmented systems that fail to meet the needs of marginalized populations [11,15]. Addressing these gaps is crucial to ensuring digital health advancements benefit all patients, including those with disabilities.

Given the diverse experiences and needs of PwPD in navigating virtual care, understanding their specific challenges and preferences is essential for optimizing healthcare delivery. This study investigated the utilization of virtual care and digital health access among PwPD compared to those without chronic conditions (NCC). Specifically, it examined usage rates, preferred modalities, the impact of interoperability, and the role of access to personal digital health information.

The study sought to answer the following research questions: How do digital health systems and the accessibility of personal health information (PHI) affect patients with physical disabilities compared to those without disabilities? How does the utilization and perception of preference for virtual care modalities differ between patients with physical disabilities and those without disabilities?

By exploring these questions, this research aimed to identify actionable insights to improve digital health access and virtual care delivery for PwPD, ultimately contributing to more equitable and effective healthcare systems.

## Materials and methods

Study design

The study design used a retrospective, cross sectional survey methodology from the 2022 Canadian Digital Health Survey (CDHS)[16]. 

Measurement tool and participants

The measurement tool was a self-completed web-based survey designed by Canada Health Infoway and delivered by Leger to Canadians over the age of 16 via Computer-Assisted Web Interviewing technology (CAWI). The survey comprised different sections. However, this study used data from four sections including demographics, health status and utilization, personal use of digital health and value, access to personal health information and access to virtual care services (see Appendix). The primary objective of this survey is to provide updated information on Canadians' attitudes, use, perceptions, and expectations of digital health services in Canada. The survey took about 20 minutes to complete. The results from surveyed individuals who stated they had a physical disability that is expected to last or has already lasted six months or more were compared to individuals who do not have any chronic illness (NCC). The inclusion criteria were any Canadians over the age of 16 who have used healthcare in Canada over the 12 months preceding the survey. In addition, the participants had to be proficient in English or French and had to have access to an electronic device that could connect to the internet. To identify the respondents with PwPD, the data was filtered using the respondents who selected physical disability for question 6 of the survey, “Do you have any of the following conditions or chronic illness diagnosed by a health professional” (a condition that is expected to last or has already lasted six months or more). To identify the respondents with NCC, the data was filtered from respondents who selected that they do not have a chronic illness from the same question (see Appendix). Patients who had other chronic conditions but no physical disability were excluded from the comparison given the focus was exclusively on persons with physical disabilities.

Data analysis

All data was processed using SPSS 29 (IBM Corp., Armonk, NY, USA) using the data file that was delivered by Leger.

We used the independent t-test to compare PwPD and NCC for continuous variables. We used the chi-square to compare dichotomous variables and missing values were excluded. Pearson correlation was used to compare age with video consults and telephone consults.

Ethics approval

Under Article 2.4 of the TCPS2, research that relies exclusively on the secondary use of anonymous information does not require ethics review. The Research Ethics Board at the Toronto Metropolitan University (TMU) determined that a review was not required.

## Results

Demographics

The survey had 12,455 respondents from across Canada (see Table 1). Six hundred seventy-four (674) PwPD and 5273 NCC were identified. Six thousand five hundred eight (6,508) respondents were excluded for having other chronic conditions.

**Table 1:** Demographics, 2022 Canadian Digital Health Survey, patients with physical disabilities (PwPD) vs those without chronic conditions (NCC) Offers a comprehensive comparative analysis of the demographic and socioeconomic characteristics distinguishing individuals with physical disabilities from those without chronic conditions. The data encompasses sample sizes, corresponding percentages, and the outcomes of statistical tests to assess significant differences between the two groups.

Variables	Physical Disability	No Chronic Conditions	X^2 ^or F (df)	P value
Gender, n (%)			6.839 (1)	.009
Male	349 (51.8)	2448 (46.5)		
Female	324 (48.1)	2815 (53.5)		
Age, n (%)			229.368 (7)	< .001
16 to 24 years	13 (1.9)	539 (10.2)		
25 to 34 years	45 (6.7)	957 (18.1)		
35 to 44 years	74 (11.0)	1019 (19.3)		
45 to 54 years	137 (20.3)	918 (17.4)		
55 years and older	405 (60.1)	1840 (34.9)		
Mean (SD)	57.14 (14.4)	46. 67 (16.6)		
Income, n (%)			2.209 (4132)	<0.001
0-$24,999	124 (18.4)	225 (4.3)		
$25,000-$49,999	124 (18.4)	550 (10.4)		
$50,000-$79,999	91 (13.5)	770 (13.3)		
$80,000-$99,999	68 (10.1)	704 (13.4)		
$100,000-$149,999	59 (8.8)	819 (15.5)		
$150,000-$3,750,000	39 (5.8)	631 (11.9)		
Area, n (%)			.391 (1)	.532
Rural	81 (12)	591 (11.2)		
Other	593 (88)	4682 (88.8)		

The PwPD group was older at 57.14 years (±SD=14.4) compared to 46. 67 years (±SD=16.6) in NCC. The mean income in PwPD was $73,000 (±SD =1.59) per year, $25,000 (±SD=1.54) less than NCC. Age had a negative correlation with virtual video consult for both groups at r= -0.226 (n=438, p=<0.001) for PwPD and r= -0.195 (n=2262, p=<0.001). Conversely, age has a positive correlation with the use of a telephone for both groups at 0.143 (n=438, p=0.003) for PwPD and 0.116 (n=2262, p=<0.001). 

Use of digital health services

The respondents were asked if they were interested in various digital health services. PwPD were slightly less interested in ebooking doctor appointments ( 66.9%, n=451) compared to NCC (74.9%, n=3947, X2=10.570, p=.005). Interest in having a telephone consultation with a healthcare provider, 74.2% (n=500) of PwPD stated yes vs NCC at 69.5% (n=3664, X2=7.824, p=.020). PwPD were more interested in having a prescription sent directly to a pharmacy by their physician (PwPD 84.3%, n=568), compared to NCC (80.9%, n=4264, X2=6.977, p=.031).

84.9% (n=231) of PwPD stated that they used digital health services in the last 12 months (see Table 2), vs 76.3% (n=1243) for NCC. PwPD had their prescription sent directly to the pharmacy 12.4% more (92.1%, n=396) vs NCC (79.7%, n=1764). The rate of telephone consults was 17.4% more often with PwPD (85.5%, n=342, NCC=68.1%, n=1357). Consultation with a healthcare provider (HCP) via email/SMS or chat was also 17.4% greater in the PwPD group (85.2%, n=109; NCC: 67.8%, n=346). When asked if they took part in a remote patient monitoring (RPM) using a device to manage a chronic health condition at home and transmit the results to a healthcare provider so that progress could be monitored, PwPD had 15.4% more usage (74.7%, n=59; NCC: 59.3%, n=147). 

**Table 2:** Digital Health Services Used in the Past 12 Months Analyzes healthcare technology utilization between individuals with physical disabilities and those without chronic conditions. It examines eight areas of digital health engagement, including access to electronic health records and telemedicine services. Each category provides response distributions along with sample sizes and percentages for both groups. Chi-square tests identify significant differences in utilization patterns, highlighting how physical disability impacts engagement with modern healthcare delivery. PHI: personal health information, HCP: healthcare provider, RPM: remote patient monitoring

Variables	Physical Disability	No Chronic Conditions	X^2 ^or F (df)	P value
Accessing PHI, n (%)			10.570 (2)	0.005
Yes	231 (84.9)	1243 (76.3)		
No	38 (5.6)	370 (7)		
Don’t Know	3 (0.4)	16 (0.3)		
Make an appointment with MD, n (%)			8.091 (2)	18
Yes	147 (85.5)	778 (75.6)		
No	478 (70.9)	4070 (77.2)		
Don’t Know	24 (3.6)	174 (3.3)		
Viewing electronic referral to specialist, n (%)			5.725 (2)	0.057
Yes	74 (78.7)	348 (69.2)		
No	18 (2.7)	151 (2.9)		
Don’t Know	2 (0.3)	4 (0.1)		
Consult with HCP by email/SMS/chat, n (%)			17.030 (2)	< .001
Yes	109 (85.2)	346 (67.8)		
No	17 (13.3)	160 (31.4)		
Don’t Know	2 (1.6)	4 (0.8)		
Phone Consult with HCP, n (%)			50.283 (2)	< .001
Yes	342 (85.5)	1357 (68.1)		
No	54 (13.5)	616 (30.9)		
Don’t Know	4 (1)	20 (1)		
Used RPM to monitor and transmit info, n (%)			8.914 (2)	12
Yes	59 (74.7)	147 (59.3)		
No	17 (21.5)	97 (39.1)		
Don’t Know	3 (3.8)	4 (1.6)		
Prescription sent directly to pharmacy, n (%)			38.970 (2)	< .001
Yes	396 (92.1)	1764 (79.7)		
No	29(6.7)	423 (19.1)		
Don’t Know	5 (1.2)	26 (1.2)		
Accessing clinical notes of a medical encounter with HCP, n (%)			6.852 (2)	0.032
					Yes	113 (81.9)	389 (70.9)		
No	24 (17.4)	155 (28.2)							
Don’t Know	1 (0.7)	5 (0.9)							

Results from having access to PHI (see Table 3) have shown that people strongly agree they can set and make progress toward health goals due to access to PHI, PwPD at 29% (n=69), and NCC at 24.6% (n=323). In addition, 28.7% (n=64) of PwPD and 23.9% (n=294) of NCC strongly felt they could avoid at least one visit with a doctor due to having access to PHI. However, when asked if having access to PHI avoided an ER visit at least once, the PwPD group showed 22.8% (n=42) and 15.8% (n=158) for NCC. There was no statistical difference between the PwPD and the NCC respondents.

**Table 3:** Impact/Benefit of Accessing Personal Health Information Presents a comparative analysis of self-reported healthcare behaviors influenced by access to personal health information (PHI). It contrasts responses from individuals with a physical disability against a control group with no chronic conditions. The table assesses the impact of PHI access on three key outcomes: 1) the ability to set and track health goals, 2) the ability to avoid an in-person visit with a medical doctor (MD), and 3) the ability to avoid an in-person emergency room (ER) visit. Responses are captured on a four-point Likert scale (Strongly disagree to Strongly agree). Statistical analysis using Chi-square (χ²) tests is included to determine if there are significant differences in these behaviors between the two groups. The P-values indicate that there are no statistically significant differences found for any of the three variables.

Variables	Physical Disability	No Chronic Conditions	X^2 ^or F (df)	P value
Able to set and make progress towards health goals due to access of PHI, n (%)			3.295 (1)	0.348
Strongly disagree	19 (8)	141 (10.5)		
Moderately disagree	45 (18.9)	239 (17.7)		
Moderately agree	105 (44.1)	635 (47.1)		
Strongly agree	69 (29)	332 (24.6)		
Able to avoid an in-person visit with my MD at least 1x due to access of PHI, n (%)			3.622 (2)	0.305
Strongly disagree	50 (22.4)	285 (23.2)		
Moderately disagree	44 (19.7)	301 (24.5)		
Moderately agree	65 (29.1)	351 (28.5)		
Strongly agree	64 (28.7)	294 (23.9)		
Able to avoid an in-person ER visit at least 1x due to access of PHI, n (%)			6.125 (3)	0.106
Strongly disagree	66 (35.9)	367 (36.6)		
Moderately disagree	35 (19)	233 (23.3)		
Moderately agree	41 (22.3)	244 (24.4)		
Strongly agree	42 (22.8)	158 (15.8)		

When asked about the various forms of digital health they can access (see Table 4), the list of medication was accessible by 39.7% (n=108) of PwPD vs 34.4% (n=561) of NCC, patient visit summary was 26.1% (n=71) in PwPD, 17.8% (n=290) in NCC, and specialist notes 24.6% (n=67) PwPD vs 17.6% (n=287) NCC.

**Table 4:** Digital Health Access Provides a comparative analysis of patients' ability to electronically access three specific types of their health information. It contrasts the access rates between individuals with a physical disability and a control group with no chronic conditions. The three types of health information studied are: 1) a list of current medications and medication history, 2) patient visit summaries, and 3) specialist consultation notes or records. The table presents the number and percentage of individuals in each group who can or cannot access this information electronically. Statistical analysis using Chi-square (χ²) tests is included, along with P-values, to determine whether there are significant differences in access rates between the two groups.

Variables	Physical Disability	No Chronic Conditions	X^2 ^or F (df)	P value
List of your current medications and medication history -can currently access electronically, n (%)			2.836 (1)	0.92
No	164 (60.3)	1068 (65.6)		
Yes	108 (39.7)	561 (34.4)		
Patient visit summary -can currently access electronically, n (%)			10.439 (1)	0.001
					No	201 (73.9)	1339 (82.2)		
Yes	71 (26.1)	290 (17.8)							
Specialist consultation notes / records -can currently access electronically, n (%)			7.567 (1)	0.006
					No	205 (75.4)	1342 (82.4)		
Yes	67 (24.6)	287 (17.6)							

Analysis on digital health interoperability over the past 12 months (see Table 5) shows that communication issues between HCP delayed care, PwPD 24.4% (n=153) and NCC 12.5% (n=575). HCP missing essential parts of health records occurred 20% (n=129) of the time with PwPD and 8.1% (n=376) with NCC. 11.8% (n=75) of PwPD and 5.7% (n=262) of NCC had to redo a diagnostic test because the results were unavailable to the HCP. 20.3% (n=130) of PwPD felt that time was wasted due to a lack of shared information or communication issues, and NCC was at 10.3% (n=478). Health was compromised due to HCP communication in 14.8% (n=94) of the PwPD population and 5% (n=233) in NCC. 31.8% (n=203) of the PwPD respondents experienced frustration with repeating information to different HCP; in comparison, NCC felt that way in 15.5% (n=717) of the group.

**Table 5:** Digital health issues In the past 12 months Provides a comparative analysis of negative healthcare experiences resulting from communication and information-sharing issues between healthcare providers (HCPs). It contrasts the experiences of individuals with a physical disability against a control group with no chronic conditions over the past 12 months. The table examines six key problem areas: delayed care, missing health records, redundant diagnostic tests, wasted time, compromised health, and frustration from repeating information. For each variable, it presents the number and percentage of respondents who experienced the issue. Statistical analysis using Chi-square (χ²) tests and corresponding P-values are included to determine if there are significant differences in the frequency of these negative experiences between the two groups.

Variables	Physical Disability	No Chronic Conditions	X^2 ^or F (df)	P value
Communication issue between HCP delayed care, past 12 months, n (%)			80.055 (3)	< .001
Yes	153 (24.4)	575 (12.5)		
No	404 (64.4)	3652 (79.5)		
Don’t Know	64 (10.2)	319 (6.9)		
Prefer Not to Answer	6 (1)	48 (1)		
HCP missing important parts of health record, past 12 months, n (%)			105.233(2)	< .001
Yes	129 (20)	376 (8.1)		
No	427 (66.3)	3777 (81)		
Don’t Know	83 (12.9)	467 (10)		
Prefer Not to Answer	5 (0.8)	43 (0.9)		
Redo a diagnostic test due results not available to HCP, n (%)			45.338 (3)	< .001
Yes	75 (11.8)	262 (5.7)		
No	512 (80.4)	4079 (88.4)		
Don’t Know	46 (7.2)	216 (4.7)		
Prefer Not to Answer	4 (0.6)	55 (1.2)		
Time was wasted because HCP communication or share information, n (%)			62.945 (3)	< .001
Yes	130 (20.3)	478 (10.3)		
No	447 (69.8)	3801 (81.9)		
Don’t Know	53 (8.3)	316 (6.8)		
Prefer Not to Answer	10 (1.6)	44 (0.9)		
Health was compromised due to HCP communication, n (%)			113.808 (3)	< .001
Yes	94 (14.8)	233 (5)		
No	465 (73.3)	4061 (87.4)		
Don’t Know	61 (9.6)	301 (6.5)		
Prefer Not to Answer	14 (2.2)	49 (1.1)		
Frustrated repeating information to different HCP, n (%)			110.361 (3)	< .001
Yes	203 (31.8)	717 (15.5)		
No	396 (62.1)	3680 (79.4)		
Don’t Know	28 (4.4)	190 (4.1)		
Prefer Not to Answer	11 (1.7)	47 (1)		

Virtual care

An analysis of virtual care (VC) utilization reveals significant differences between patients with PwPD and those with NCC, highlighting varying patterns of engagement with digital health services.

T-test showed PwPD respondents were more likely to visit a doctor in-person vs virtually on average (mean=4.78 visits in-person, SD=8.6, p=<.001, 2.23 visits virtually, SD=3.9, p=<.001, n=674) in comparison to NCC (mean=1.17 visits in-person, SD=3.2, .63 visits virtually, SD=1.54, n=5273, p=<.001) over the past 12 months.

The average number of visits to an ER/Urgent Care in the past 12 months was higher for PwPD (mean=1.13 visits in-person, SD=2.99, 0.28 virtually, SD=3.92 n=674, p=<.001) in comparison to NCC (mean=0.32 visits in-person, SD=1.25, 0.06 virtually, SD=.452, n=5273, p=<.001).

Regarding specialists, 18.7% of the PwPD (n=82, X2=50.749, p=<.001) respondents reported their most recent virtual visit was with a specialist, 9% more than NCC (9.7%, n=219).

The telephone was offered and used more recently than any other modality for virtual care for both groups. The telephone was offered 82.2% (n=360) in PwPD and 77% (n=1749) in NCC.

Reasons for choosing virtual care, it saved money on transportation, parking, care for dependents, no time off work (PwPD 27.9 % n=122, NCC 16.5%, n=373, p=<.001), reduced wait times (PwPD 29.7 %, n=130, NCC 24.9%, n=566, p=.034), and virtual care was the appropriate option (PwPD 27.4 %, n=120, NCC 23.2 %, n=525, p=.060).

When asked which VC modality they would use for types of visits with MD, in-person was the top choice for routine exams (PwPD: 76.7%, n=517; NCC: 79.6%, n=4196), mental health (PwPD: 62.8%, n=423; NCC: 68%, n=3592) and sexual health (PwPD: 49.9%, n=336; NCC: 50%, n=2634). The telephone was the top choice for follow-ups for prescription renewal (PwPD: 61.1%, n= 412; NCC: 50.6%, n=2670), minor health problems (PwPD: 52.7%, n=355; NCC: 41.6%, n=2195) and follow-ups (PwPD: 46.1%, n=311; NCC: 37.5%, n=1976).

## Discussion

Digital health adoption and utilization

The utilization data from the CDHS examined health status and utilization, personal use of digital health and value, access to PHI and access to virtual care services. It revealed substantially higher engagement across multiple digital health services among PwPD, with notably 17.4% higher telephone consultations (85.5% vs 67.8%), 17.4% higher email/SMS consultations (85.2% vs 67.8), and 12.4% higher rates of electronic prescriptions (92.1% vs 79.7%) compared to NCC. Remote patient monitoring showed a 15.4% higher utilization rate among PwPD (74.7% vs 59.3%), indicating greater engagement with chronic disease management tools. This enhanced utilization of digital services suggests that PwPD are actively engaging with and potentially benefiting from the expanding digital health ecosystem.

The data demonstrates significantly higher overall access to PHI among PwPD (84.9%) compared to NCC (76.3%). Access to PHI yielded positive outcomes for both groups, with comparable proportions reporting improved ability to set and achieve health goals (PwPD: 73.1%, NCC: 71.7%). Notably, 28.7% of PwPD and 23.9% of NCC reported avoiding at least one in-person doctor visit due to PHI access, while 22.8% of PwPD and 15.8% of NCC reported avoiding an emergency room visit, suggesting significant potential for reducing unnecessary healthcare utilization [8].

However, our findings also revealed important challenges in using digital health services. PwPD reported significantly higher rates of care delays due to communication issues (24.4% vs NCC 12.5%), missing health records (20% vs NCC 8.1%), and repeated diagnostic tests (11.8% vs NCC 5.7%). These challenges were particularly pronounced in the PwPD group, with 31.8% reporting frustration with repeating information to different healthcare providers compared to 15.5% of NCC. These findings suggest that digital health must be thoughtfully integrated into care plans with appropriate safeguards to prevent adverse outcomes and ensure effective communication between patients and healthcare providers is preserved.

Our data suggests that while digital health services offer significant benefits for PwPD, there remains a critical need to address interoperability challenges and optimize service delivery to meet the specific needs of this population. The higher reported rates of communication issues and care delays among PwPD highlight the importance of developing more robust and integrated digital health systems that can effectively support complex chronic disease management.

Regarding the interoperability issue across Canada, healthcare institutions, clinicians, vendors, and the government must invest and work together to allow for a smoother transfer of patient data.

Virtual care

Our findings demonstrate substantially higher virtual care utilization patterns among PwPD compared to NCC, with approximately three-and-a-half-fold higher virtual visits (2.23 vs. NCC 0.63) over a 12-month period and a four-fold higher in-person visits (4.78 vs. NCC 1.17). This marked difference in healthcare access patterns underscores the complex care needs of the PwPD population and their increased engagement with healthcare services.

Regarding virtual care preferences, PwPD showed significantly higher interest in telephone consultations (74.2% vs 69.5%, p=.020). Telephone emerged as the preferred modality for specific healthcare needs, particularly for follow-up appointments (46.1% vs NCC 37.5%), prescription renewals (61.1% vs NCC 50.6%), and minor health issues (52.7% vs NCC 41.6%). However, in-person visits remained the preferred choice for routine examinations (76.7%, NCC 79.6%), mental health consultations (62.8% vs NCC 68.1%), and sexual health concerns (49.9%, NCC 50%). The selection of virtual care was primarily driven by practical considerations, including reduced transportation costs and shorter wait times (29.7% PwPD vs 24.9% NCC, p=.034). These findings suggest that virtual care modalities serve as effective complementary services in managing PwPD, particularly in reducing barriers related to transportation and associated costs, sentiments shared by other researchers [17]. Furthermore, the preference for telephone-based consultations indicates the importance of maintaining simple, accessible communication channels for this population [18].

Potential cost savings 

In the current study, the PwPD group had a higher proportion of visits in-person or virtually in the last 12 months. PwPD had 2.8 times more in-person visits with doctors and 3.5 times more ER visits than NCC. For virtual visits with the doctor, they had 3.5 times more visits and 4.6 times more visits than NCC. The potential cost savings for the Canadian health system from using virtual care services to support care for PwPD are enormous. Based on Canada's population of 38 million and the survey's response rate of 5.4% for PwPD, approximately 2 million Canadians are estimated to have physical disabilities. If even 10% of this group (200,000 individuals) were to avoid one doctor appointment in the next 12 months, with each visit costing $56.02, the potential annual savings could amount to $11 million. This calculation illustrates the significant financial impact that increased virtual care utilization could have on the healthcare system. While the numbers above are conservative calculations on the potential savings, the general implications are clear. Using the same estimates for emergency room visits, the potential savings would be higher since the PwPD group also stated that they were able to avoid at least one ER visit in 45.1% of the population. The average cost of one ER visit in Canada was $158, using the same estimate of 2 million, 900,000 would have visited the ER one fewer time, with a cost savings of over $142 million to the system [19].

In addition, 12% of the PwPD group reported having to redo a diagnostics test. If the assumption is made that the most common blood test, CBC, which is also one of the cheapest tests to perform at $5, was not repeated 12% of the time, it would result in a savings of $1.2 million. If we were to extrapolate more realistic numbers and include the actual costs of performing the blood draw, staffing and equipment at $15.62 and increase the cost of the CBC to the national average of $10.96, the savings would translate to $6.3 million [20]. The reality is that the saving potential is far greater since most patients do not get one singular blood test, and there are other more expensive tests, such as X-rays.

In addition to the direct cost savings to the healthcare system, the suggested improvements would also positively impact the patient financially by reducing income losses by avoiding travel and dependent care expenses. Studies have shown that the population of PwD continues to rise and while the PwPD represents a smaller segment of this larger group, there are savings implications [21]. Similarly other studies have indicated that the main reasons for choosing virtual care are convenience and financial reasons. In the current study, PwPD participants had an 11% greater response to the statement “virtual care saves money” relative to the NCC counterparts. The ability to save money in a population group with lower-income earners should play a factor in what services to offer specific populations to allow equitable care.

The finding of 29.5% of the PwPD population having primary or joint responsibility for providing care for another person, 10% more than NCC, could play a role in the desire to use digital health. When these individuals have in-person visits, they will have the extra task of finding coverage for their dependents, which adds to the already high costs for their travel for an in-person doctor's visit [22].

The PwPD group is older, with a higher proportion of people in the greater 55-year-old range (60.1% vs 34.9%), which could result in issues with how they handle various technologies. The negative correlation between age and virtual video usage shows that the younger you are, the more likely you are to use virtual video, which strongly correlates with what Reid et al. found in their study [12]. Telephone usage had the opposite results of video, showing that the older you are, the more likely you are to use the telephone for virtual care follow-up appointments, minor health-related problems and prescription renewals, which has been found in other studies [23,24]. Other studies have shown no preference for video or telephone use [7].

With the PwPD group making $25,000 less than NCC, they will have less disposable income to spend on technology. In addition, with less income, they would be more likely to hold on to older technology which may cause more significant issues with the compatibility of accessing various digital health services. The ability to save money in a population group with lower-income earners should play a factor in the determination of healthcare service delivery to ensure that equitable care remains a key priority. 

Patients with PwPD use the services of specialists more frequently; they reported 9% more for recent visits, and accessing specialists' notes 7%, relative to the NCC group. Specialists have to be mindful of this fact and should keep this in mind when considering which types of virtual care and digital health options to offer.

Thirty percent of PwPD choose reduced wait times as the primary factor for virtual care. The reduced wait times allow people with PwPD to be comfortable in their homes and to get on with their lives faster post-encounter; they can return to their job with little interruption. In addition, there is no commute to deal with, allowing these patients to be more comfortable within their homes and minimize discomfort [25].

PwPD and NCC feel in-person visits are more popular with the confidential type of appointments. However, telephone consults are better suited for minor issues and have worked well for history-taking and follow-ups [26]. This information could help steer HCP on when to offer specific VC modalities to certain cases. 

With the change in fee structures for virtual care services in Ontario, after this survey was conducted, it will be essential to see the impact those cuts have on all aspects of virtual care [27].

Based on the research, the recommendation to healthcare institutions and clinicians around Canada is to identify patients with PwPD and allow them to choose the modality for their appointment if it is deemed appropriate, especially for minor issues.

HCP need to be much more conscious of which patients in their rosters have PwPD and adjust their workflows to allow for the opportunity for future healthcare encounters to occur at least using the telephone if the visit enables it. For example, at a bare minimum, if a follow-up is required for minor health or prescription renewal, a telephone consult should be offered. In addition, if the patient with PwPD is responsible for other individuals in their household, every effort should be made to accommodate a virtual care appointment, if appropriate.

A possible solution for HCP would be that when a patient with PwPD is booking an appointment with them, the booking system could flag or notify the person booking that the patient has a PwPD or a PwPD with other dependents and give the patient the choice of a telephone or virtual consultation or to come into the office. The telephone is a simple medium that has been around for a long time, and it would take a minimal amount of work with little to no additional costs to incorporate it into the current workflow, which was readily observed during the pandemic. The popularity of the telephone has been discussed throughout the paper. With the telephone being the number one method offered, clinicians need to consider adding other options to give a better balance of services to all patients if applicable. One recommendation for clinicians is to look at their various virtual care offerings to see if there are any hurdles and potential performance enhancements to make those more accessible to all populations without compromising care or security. Some examples would be a simplified signup process, a login process with fewer steps and making the user interface more user-friendly. Similar solutions should be considered by the vendors of these virtual care platforms. 

Limitations

This study has several noted limitations that may impact the interpretation of its findings. With online surveys as the primary data collection method, there is a risk of excluding Canadians who lack access to technology or have limited internet connectivity. This exclusion could disproportionately affect marginalized populations, leading to a less representative sample. Patients with physical disabilities may also face challenges navigating survey websites or inputting responses, introducing potential bias and limiting the generalizability of the findings.

Self-reporting was used to identify chronic conditions, and there is no way to validate the accuracy of these reports. This limitation could lead to skewed data, particularly if respondents misreport or misunderstand their medical conditions. Additionally, the severity of physical disabilities was not assessed, which could impact the results. For example, individuals with severe physical disabilities are likely to have different experiences with digital health compared to those with milder symptoms.

Outliers were identified in the income and healthcare visit frequency responses. However, these data points were not excluded, as the author determined, based on clinical and personal experience, that the responses could be accurate. While income data was not central to the study’s findings, the frequency of visits provided valuable insight into healthcare utilization patterns.

Another limitation is the lack of information on the types or ages of the technology respondents used to access digital health services. Variations in device capability or usability could influence user experiences and, consequently, the study’s results. Similarly, the sample size of rural respondents was too small to draw meaningful conclusions. Future studies should consider a larger sample of individuals with chronic conditions to explore potential differences between urban and rural populations.

The use of unweighted data also poses a limitation. Data sets analyzed in SPSS were unweighted, whereas the Excel data provided by Leger included weighting adjustments. The decision to use unweighted data was informed by prior research cautioning against weighting, as outlined in a study of seven data sets [28]. However, for future submissions, it would be beneficial to analyze both weighted and unweighted data to provide a more robust comparison.

The COVID-19 pandemic has further underscored the importance of digital health services while amplifying the challenges and disparities faced by vulnerable populations. During the pandemic, the reliance on digital health skyrocketed, highlighting both the potential and shortcomings of virtual care [29]. Many patients, particularly those with chronic conditions like PwD, were forced to adapt quickly to digital solutions. This context emphasizes the need for future studies to explore how pandemic-related shifts in healthcare delivery have affected patients’ long-term engagement with digital health technologies.

By addressing these limitations in future research, we can gain a deeper understanding of the digital health experiences of Canadians and improve the inclusivity and effectiveness of virtual care systems.

## Conclusions

This comprehensive analysis of healthcare accessibility for PwPD in Canada reveals critical insights into the current state and future trajectory of digital health integration. The findings demonstrate that PwPD, as frequent healthcare system users, serve as an optimal metric for evaluating healthcare delivery effectiveness and identifying systemic barriers. The study underscores significant challenges in digital health interoperability, with PwPD reporting heightened frustration regarding information flow between healthcare providers, ultimately affecting health outcomes. This research contributes to the growing body of evidence supporting the relationship between digital health quality and overall care outcomes in the Canadian healthcare context. A notable discovery is the marked preference for telephone consultations among virtual care modalities, suggesting the importance of familiar and accessible technology in healthcare delivery. This research opens several avenues for future investigation, particularly in understanding the optimization of digital health solutions and the nuances of virtual care preferences for vulnerable populations. Future research directions should examine the preferences for specific virtual care modalities, incorporate more inclusive data collection methodologies, and expand investigations to other chronic condition populations. Additionally, comprehensive evaluation of digital health initiatives, particularly in light of recent substantial government investments, will be crucial for optimizing healthcare delivery. These findings underscore the necessity for coordinated efforts among stakeholders to enhance digital health infrastructure and ultimately improve healthcare accessibility and outcomes for all Canadians.

## Appendices

Canadian Digital Health Survey 2022

Part I: Primary Demographics:

1. In what year were you born?

Please enter your response as a four-digit number (for example, 1977).

2.  In which province/territory do you reside?

a)     Newfoundland & Labrador

b)     Nova Scotia

c)     Prince Edward Island

d)     New Brunswick

e)     Quebec

f)      Ontario

g)     Manitoba

h)     Saskatchewan

i)      Alberta

j)      British Columbia

k)     Northwest Territories

l)      Nunavut

m)    Yukon Territory

n)     Outside Canada

3. What are the first 3 digits of your postal code?

[A0A] do not enter any spaces

4. What sex was assigned to you at birth?

a)     Female

b)     Male

c)     Prefer not to answer

5. How would you describe your gender identity? 

a)     Female gender

b)     Male gender

c)     Gender diverse

d)     Another gender, please specify - ____________

e)     Prefer not to answer

6. Do you have any of the following conditions or chronic illness diagnosed by a health professional (a condition that is expected to last or has already lasted 6 months or more.) Please select all that apply.

a)     Chronic pain

b)     Cancer

c)     Diabetes of any type

d)     Chronic obstructive pulmonary disease (COPD)

e)     Arthritis

f)      Cardiovascular disease (CVD) (e.g. coronary heart disease)

g)     Alzheimer’s disease or any other dementia

h)     Developmental disability (e.g. intellectual disability, autism)

i)      Drug or alcohol dependence

j)      Obesity

k)     Learning disability (e.g. ADHD)

l)      Emotional, psychological, or mental health conditions (e.g. anxiety, depression, bipolar disorder, substance abuse, anorexia, etc.)

m)   Physical disability

n)     Sensory disability (e.g. hearing or vision loss)

o)     Another chronic illness, please specify - _________

p)     I do not have a chronic illness

q)     Prefer not to answer

7. Do you have primary or joint responsibility for providing care and/or assistance to any of the following individuals? The assistance we’re referring to does not include employment/work done for pay. We are referring to voluntary assistance which might include taking the individual to a medical appointment, meeting with their doctor, or any other voluntary activity that supports their health and wellbeing.

a)     Yes, I do have primary or joint responsibility for providing care and/or assistance to someone

b)     No

Part II: Health Status & Utilization

8. Do you have a family doctor or regular place of care, such as a health centre or a family medical/medicine group?

a)     Yes

b)     No

c)     Don’t know

9. In the PAST 12 MONTHS, please specify how many times you have done each of the following (*Do not count times when you were an overnight patient in a hospital or if you were supporting a loved one or friend you are caring for during their visit with a health care professional).

By ‘virtually’, we are referring to a telephone, video, email / text or SMS visit (not in-person).

If you are unsure of the number of times you have done each, please provide your best guess.

a)      In-person health visits/encounters with a family doctor or regular place of care, a specialist physician, walk-in clinic/ general practitioner

b)     In-person health visits/encounters to an emergency room or urgent care center

c)      Obtained a prescription in-person

d)     Virtual health visits/encounters with a family doctor or regular place of care, a specialist physician, walk-in clinic/ general practitioner

e)      Virtual health visits/encounters to an emergency room or urgent care center

f)      Obtained a prescription virtually

PART III - Personal use of digital health and value

14.  Are you interested in having access to this digitally enabled health service, whether you currently have access or not?

15. Have you ever accessed this digitally enabled health service services at anytime in the past?

16. And did you use this service in the last 12 months?

01 - Yes

02 - No

03 - Don’t know

a)   Access your own personal health information electronically (e.g., lab test results, medication history, immunization record)

b)   Make an appointment with your regular doctor or place of care electronically (for example, on a secure website)

d)     View an electronic version of your referral to a specialist sent by your regular doctor to a specialist

e)   Visit with your health care provider virtually online by video (similar to a Zoom or Skype call - through a web-based or smart phone application)

f) Consult with a health care provider about a specific health issue or concern without having to phone or visit an office / clinic - either by e-mail or electronic text-SMS message or online chat

g)    Have a telephone consultation with a healthcare provider 

h)   Take part in a remote patient monitoring/telehomecare program using a device to manage a chronic health condition from your home and electronically transmit the results to a healthcare provider so that they can monitor your progress

i)Take part in a remote patient monitoring/telehomecare program using a device to manage symptoms related to COVID-19

j) Have a prescription sent directly to your pharmacy by your physician without receiving a paper prescription

k)   Send a request electronically for a prescription renewal

l)Access technology (e.g., websites, mobile applications (apps), telephone services, virtual services, interactive online tools) to help or support your mental health/ wellness (e.g., depression, psychosis, meditation) or substance use (e-mental health)

m)  Access websites or use mobile applications (apps) on a smartphone or digital tablet to help you monitor certain aspects of your health or well-being (e.g. your weight, your dietary habits, the quality of your sleep, your mood, your physical activity, your blood pressure, and your blood sugar level)

n)   Receive electronic reminders, notifications or alerts from any of your health care providers or places of care (e.g., an appointment reminder, medication reminders, confirmation or request to cancel, a notice of lab or diagnostic testing appointments, invitation or notice of health promotion clinics, notice of education activities or resources)

p)     Access to clinical notes associated with a medical encounter either with a primary health provider or specialist provider (i.e., a consultation report or medical notes)

PART IV - Access to Personal Health Information

You had mentioned that you have accessed your personal health information electronically, the following questions are about the online source where you accessed your personal health information.

18. Using a number from 1 to 4 (with 1 being strongly disagree and 4 being strongly agree), do you agree with the following statements about the portal where you accessed your personal health information electronically. [4-strongly agree, 3-moderately agree, 2-moderately disagree, 1-strongly disagree] The online source where I access my personal health information is easy to use

a)     Overall, I am satisfied with the online source where I can access my personal health information

b)     I feel more informed about my health as a result of accessing my personal health information online

c)     I am able to set and make progress towards health goals because I can access my personal health information online.

d)     I can better manage my health because I can access my personal health information online

e)     I was able to avoid an in-person visit with my family doctor/ regular place of care or to a walk-in clinic at least once because I was able to access my personal health information electronically

f)      I was able to avoid an in-person visit to an emergency room at least once because I was able to access my personal health information electronically

19. What specific health information can you currently access electronically? Please select all that apply.

a)   Lab test/diagnostic results

b)   Immunization history/records (including COVID-19 immunization records)

c)   List of your current medications and medication history

d)   Patient visit summary

e)   Specialist consultation notes / records

f)    Other - please specify___

PART V - Access to Virtual Care Services

You had mentioned that you visited your health care provider in the past by video, telephone, or email/text-SMS message. The following questions are about your MOST RECENT VIRTUAL ENCOUNTER that was either by video, telephone, text-SMS message, email or a combination of some or all these technologies. Note a telephone call to book an appointment/consult or COVID-19 vaccine does not apply here.

21. Was your most recent virtual health care visit mainly with: Please select one only. Your family doctor or regular place of care

a)     A general practitioner who is not your family doctor (i.e., from a virtual walk-in clinic or a doctor from a private vendor like Babylon)

b)     A nurse practitioner

c)     A specialist physician who is not your regular family doctor (e.g., cardiologist)

d)     811 or nurse telehealth line

e)     Emergency room or urgent care centre

f)      A pharmacist

g)     A mental health care provider (e.g., psychiatrist, psychologist)

h)     Other types of health providers (regulated health professionals that may include dentists, dieticians, midwifes, homeopathic doctor, etc.)

i)      Other - please specify ____

22. What was the modality (I.e., method of communication) of that most recent visit virtual visit with a healthcare provider? Please select one only.

a)     A virtual video visit

b)     A telephone consultation

c)     A messaging/email consultation/ online chat

d)     Other - please specify_______

22b. What modalities were you offered for your most recent virtual health visit/ encounter? Please select all that apply

a)     Virtual video

b)     Telephone

c)     Messaging / email / online chat

d)     In-person

e)     Other - please specify _________

f)      I don’t know

22c.  You mentioned your most recent virtual visit was via _____ [insert from Q22]. On a scale of 1 to 4 (with 1 being strongly disagree and 4 being strongly agree) do you agree or disagree with the following statements when it comes to your most recent virtual health visit / encounter?

[4= Strongly agree, 3= moderately agree, 2= moderately disagree, 1=Strongly disagree]

a)     My health concern was addressed with the virtual visit

b)     I was able to communicate my health issue(s) virtually as well as I would have at an in-person consult

c)     I felt safe (emotionally and physically) during my virtual appointment

23. What were the reasons for choosing a virtual visit instead of an in-person visit with the healthcare provider? Please select all that apply.

a)     Virtual visit was recommended by my healthcare provider

b)     Virtual care was the most appropriate option for the health concern I had

c)     It saved me time (by avoiding travel or arranging care for dependents, etc.)

d)     It saved me money (by not having to pay for transportation/parking, care for dependents, nor having to take time off work, etc.)

e)     Allowed me to access care I would otherwise not have access to due to geographic distance

f)      Allowed me to access care I would otherwise not have access to due to a physical handicap

g)     It reduces wait times

h)     It prevents me/family members from being exposed to infectious diseases at health facilities

i)      Allowed me to get treatment faster after hours

j)      Allowed me to include caregivers (e.g., family members) who otherwise would not have been able to participate in an in-person visit

k)     Other - please specify ____

## References

[1] Government of Canada, D. of F. (2023, March 28 (2023). Canadian Survey on Disability, 2017 to 2022. Chapter 2: Investing in public health care and affordable dental care: Budget.

[2] Swenor B, Deal JA (2022). Disability inclusion as a key component of research study diversity. N Engl J Med.

[3] Marks MB, Teasell R (2006). More than ramps: accessible health care for people with disabilities. CMAJ.

[4] Abernethy A, Adams L, Barrett M (2022). The promise of digital health: then, now, and the future. NAM Perspect.

[5] Kruse CS, Krowski N, Rodriguez B, Tran L, Vela J, Brooks M (2017). Telehealth and patient satisfaction: a systematic review and narrative analysis. BMJ Open.

[6] Lawford BJ, Hinman RS, Morello R, Oliver K, Spittle A, Bennell KL (2022). Perceptions about the efficacy and acceptability of telephone and video-delivered Allied Health Care for adults with disabilities during the COVID-19 pandemic: a cross-sectional national survey. Arch Phys Med Rehabil.

[7] Hammersley V, Donaghy E, Parker R (2019). Comparing the content and quality of video, telephone, and face-to-face consultations: a non-randomised, quasi-experimental, exploratory study in UK primary care. Br J Gen Pract.

[8] Kubes JN, Graetz I, Wiley Z, Franks N, Kulshreshtha A (2021). Associations of telemedicine vs. in-person ambulatory visits and cancellation rates and 30-day follow-up hospitalizations and emergency department visits. Prev Med Rep.

[9] Friedman C, VanPuymbrouck L (2021). Telehealth use by persons with disabilities during the COVID-19 pandemic. Int J Telerehabil.

[10] Filbay S, Bennell KL, Morello R, Smith L, Hinman RS, Lawford BJ (2022). Exploring experiences with telehealth-delivered allied healthcare services for people with permanent and significant disabilities funded through a national insurance scheme: a qualitative study examining challenges and suggestions to improve services. BMJ Open.

[11] Zhang X, Saltman R (2022). Impact of electronic health record interoperability on telehealth service outcomes. JMIR Med Inform.

[12] Reed ME, Huang J, Graetz I, Lee C, Muelly E, Kennedy C, Kim E (2020). Patient characteristics associated with choosing a telemedicine visit vs office visit with the same primary care clinicians. JAMA Netw Open.

[13] (2023). People with certain medical conditions and COVID-19 risk factors. https://www.cdc.gov/covid/risk-factors/.

[14] Hussein WF, Bennett PN, Pace S (2020). The mobile health readiness of people receiving in-center hemodialysis and home dialysis. Clin J Am Soc Nephrol.

[15] Jones M, DeRuyter F, Morris J (2020). The digital health revolution and people with disabilities: perspective from the United States. Int J Environ Res Public Health.

[16] Canada Health Infoway. (2023, March 29 (2023). Canadian Digital Health Survey: understanding Canadians' experiences with digital health. https://insights.infoway-inforoute.ca/2022-digital-health-survey.

[17] Haleem A, Javaid M, Singh RP, Suman R (2021). Telemedicine for healthcare: capabilities, features, barriers, and applications. Sens Int.

[18] Kaplan B (2020). Revisiting health information technology ethical, legal, and social issues and evaluation: telehealth/telemedicine and COVID-19. Int J Med Inform.

[19] CIHI. (2022, November 17 (2023). An overview of physician payments and cost per service. https://www.cihi.ca/en/health-workforce-in-canada-in-focus-including-nurses-and-physicians/an-overview-of-physician.

[20] Hale I (2015). Add to cart?. Can Fam Physician.

[21] Jehn A, Zajacova A (2019). Disability trends in Canada: 2001-2014 population estimates and correlates. Can J Public Health.

[22] Neven A, Ectors W (2023). “I am dependent on others to get there”: mobility barriers and solutions for societal participation by persons with disabilities. Travel Behav Soc.

[23] Abdallah L, Stolee P, Lopez KJ, Whate A, Boger J, Tong C (2022). The impact of COVID-19 on older adults’ perceptions of virtual care: qualitative study. JMIR Aging.

[24] Buawangpong N, Pinyopornpanish K, Pliannuom S, Nantsupawat N, Wiwatkunupakarn N, Angkurawaranon C, Jiraporncharoen W (2024). Designing telemedicine for older adults with multimorbidity: content analysis study. JMIR Aging.

[25] Ehde DM, Jensen MP, Engel JM, Turner JA, Hoffman AJ, Cardenas DD (2003). Chronic pain secondary to disability: a review. Clin J Pain.

[26] Glauser W (2020). Virtual care is here to stay, but major challenges remain. CMAJ.

[27] Farooqui Farooqui, S. (2022, December 12 (2023). Drop in OHIP funding shakes up Virtual Care Services as one company starts charging subscription fees. https://www.theglobeandmail.com/canada/article-ohip-virtual-health-care-funding/.

[28] Haddad C, Sacre H, Zeenny RM, Hajj A, Akel M, Iskandar K, Salameh P (2022). Should samples be weighted to decrease selection bias in online surveys during the COVID-19 pandemic? Data from seven datasets. BMC Med Res Methodol.

[29] Kfrerer ML, Zhang Zheng K, Austin LC (2024). From 0-50 in pandemic, and then back? A case study of virtual care in Ontario pre-COVID-19, during, and post-COVID-19. Mayo Clin Proc Digit Health.

